# Morphological Correlates of KIT and PDGFRA Genotypes in Gastrointestinal Stromal Tumour

**DOI:** 10.5146/tjpath.2019.01470

**Published:** 2020-05-15

**Authors:** Valli Priya, Niraj Kumari, Narendra Krishnani

**Affiliations:** Department of Pathology, Sanjay Gandhi Post Graduate Institute of Medical Sciences, Lucknow, India

**Keywords:** Gastrointestinal stromal tumours, Morphology, KIT, PDGFRA, Sequence analysis

## Abstract

***Objective:*** The aim of the study was to study the clinicopathological and immunohistochemical features of gastrointestinal stromal tumours and correlation with KIT/PDGFRA mutations.

***Material and Method:*** Eighty consecutive resected cases were genotyped for KIT exons 11, 9, 13, 17 and PDGFRA exons 18, 14, 12 and correlated with histomorphology by nonparametric tests.

***Results:*** Forty-seven cases (58.8%) were in the high-risk group. Males had higher rates of KIT exon 11 and PDGFRA exon 18 mutations than females (p=0.03). KIT and PDGFRA mutation frequencies were lower (58.8%) than western data showing KIT exon 11 mutation in 63.8%, KIT exon 9 mutation in 19% and PDGFRA exon 18 mutation in 17% of the cases. Extragastrointestinal stromal tumours (n=6) showed 100% mutation. KIT exon 11 deletion was associated with gastric location (60%) (p=0.04), spindle cells (63.3%), and high-risk stratification (66.6%) (p=0.01) while KIT exon 9 mutation was common in small intestine (66.7%) (p=0.04), in higher risk groups (66.7%) (p=0.01) and 75% of codon 502-503 duplications (p=0.03). PDGFRA 18 mutation was common in males (p=0.03), in gastric location (62.5%) (p=0.04), in cases showing mild to moderate atypia (62.5%) (p=0.01) and lower risk stratification (62.5%) (p=0.01). KIT/PDGFRA mutations were significantly associated with gender (p=0.03), location (p=0.04), nuclear atypia (p=0.01) and risk stratification (p=0.01).

***Conclusion:*** Morphological features and anatomic location may be useful in deciding molecular testing strategy, particularly in resource-limited settings, when a plethora of targetable mutations are present. An algorithm may be derived for genotyping with KIT exon 11 and PDGFRA exon 18 heading the list of targetable mutations. This approach may reduce financial burden on patients as well as workload on hospital staff.

## INTRODUCTION

Gastrointestinal stromal tumour (GIST) is the most common mesenchymal neoplasms of the gastrointestinal tract (GIT) that arises from interstitial cells of Cajal (1). Morphologically, GISTs can be of spindle cell, epithelioid, or mixed phenotype that expresses CD117 (KIT) on immunohistochemistry. These tumours also express DOG1 and CD34 (2). Mutational analysis of GISTs have shown mutations in KIT mostly occurring in exon 11 followed by exon 9 and in PDGFRA exon 18 (3). KIT exon 11 mutations include deletions, point mutations and insertions. Certain morphological features are associated with KIT 11 deletions such as gastric location, spindle cell morphology and large tumour size (4,5). KIT 11 insertions are also associated with spindle cell morphology but are generally seen in non-gastric locations (6). GISTs harbouring PDGFRA mutations are common in gastric, omental and mesenteric tumours showing nuclear pleomorphism, epithelioid morphology, plasmacytoid cells and multinucleated giant cells (7). Understanding the molecular and morphological correlations is important and may help to prioritize the sequential testing for GIST mutations, particularly in a resource-limited setting in developing countries. This study aims to compare the histomorphological and immunohistochemical features with various genotypes.

## MATERIALS and METHODS

Eighty consecutive resected GISTs received within a period of 8 years were included in the study. Clinical findings and follow-up data were retrieved from the hospital records. All cases were reviewed for tumour size, location, histological type, cellularity, presence of atypia, necrosis, mitosis, secondary changes, lymph node and distant metastasis. Risk stratification was done according to Miettinen’s classification (8). Extraintestinal GIST (EGIST) was defined by a combined approach of radiological, operative, gross and microscopic examination where the bulk of the tumour was outside the gastrointestinal tract and did not show a clear cut connection with the bowel or stomach wall. Immunohistochemistry was performed for CD117 (DAKO, Denmark), DOG1 (Novocastra, United States), CD34 (DAKO, Denmark), SMA (DAKO, Denmark), S100 (DAKO, Denmark), desmin (DAKO, Denmark) and vimentin (DAKO, Denmark).

### Molecular Analysis

DNA extraction was done from formalin-fixed paraffin-embedded tissue using the QiaAmp FFPE kit. Polymerase chain reaction (PCR) was performed for KIT (exons 11, 9, 13, 17) and PDGFRA (exons 18, 14, 12). Briefly, each PCR reaction was done in 25 μl volume using 250 ng of DNA on the ABI SimplyAmpTM thermal cycler followed by Sanger sequencing on the ABI 3130 Genetic Analyzer.

### Statistical Analysis

Categorical variables were correlated using the Chi square test and Fisher’s exact test where appropriate. Recurrence-free survival was analysed using Kaplan-Meier log-rank analysis. SPSS version 16 was used. A p value of < 0.05 was considered significant. The study was approved by the institute’s ethics committee.

## RESULTS

### Demography

Median age at presentation was 54 years (range =16-80 years) with a male to female ratio of 2:1. There were 38 gastric (47.5%), 33 small intestinal (41%), 3 colonic (4%), 4 mesenteric (5%) and 2 retroperitoneal (2.5%) cases. In the small intestine, 10 cases were in the duodenum, 17 in the ileum and 6 in the jejunum. All patients were symptomatic at presentation with presence of abdominal mass, gastrointestinal bleeding or abdominal pain.

### Histopathology

The majority of the gastric (89.5%), small intestinal (90.9%) and EGIST cases (83.3%) showed moderate to high cellularity with colonic GISTs having conspicuously low cellularity (66.7%), (p=0.15). The spindle cell type (n=52, 65%) was predominantly present in all locations, followed by mixed (n=20, 25%) and epithelioid cell type (n=8, 10%). Multinucleated tumour giant cells were seen scattered in 2 cases. The most common pattern was that of fascicles (n=63, 78.7%) followed by palisading (10 cases, 12.5%) and storiform (8 cases, 10%) (p=0.15). Secondary changes included cystic degeneration and skenoid fibres in 11 cases each (13.7%), of which 10 (12.5%) were present in the small intestine, hyalinization (8 cases, 10%), myxoid degeneration (7 cases, 8.7%), paranuclear vacuolization (3 cases, 3.7%), calcification (1 case, 1.2%), congested vessels (4 cases, 5%) and significant haemorrhage (6 case, 7.5%).

The extent of tumour infiltration was limited to the submucosa in one case and muscularis propria in 9 cases. Exophytic GISTs (n=15) involving the serosa alone were seen in 5 and serosa to muscularis propria in 10 cases. Twenty-nine cases (36.2%) had entire thickness involvement with ulceration of overlying mucosa.

Overall CD117 and DOG-1 immunoreactivity were 93.8% (n=75) and 91.3%, respectively, with high concordance between the two markers; excluding five cases (6.25%) with the CD117 (+)/DOG1(-); three (4%) cases with CD117(-)/DOG1(+) and two cases (2.5%) with CD117 (-)/DOG1(-) immune profile. The latter two cases were also KIT/PDGFRA wild type and the diagnosis was based on characteristic histomorphology, anatomical association with the gastrointestinal tract, presence of CD34 and absence of diffuse and strong expression of other immunohistochemical markers. The detailed gross, histological and immunohistochemical features are mentioned in Table 1.

**Table 1:** Gross, histological and immunohistochemical features of GIST at different locations.

**Features**		**Total (n=80)**	**Stomach (n=38)**	**Small intestine (n=33)**	**Large intestine (n=3)**	**EGIST** **(n=6)**
**Gross**						
Size (cm) mean ±Standard Deviation (Minimum-Maximum)		10.3 ± 6.28 (1.5-30)	9.8 ± 5.1 (1.5-21)	9.8 ± 6.3 (2-25)	17 ± 11.2 (10.1-30)	12.9 ± 8.9 (2.5-28)
Mucosal ulceration (n, %)		38 (475)	18 (47.4)	19(57.6)	1(33.3)	0
Necrosis (n, %)		19 (23.7)	11 (29)	5 (15.1)	1 (33.3)	2 (33.6)
Cystic degeneration (n, %)		12 (15)	6 (15.8)	5 (15.1)	1 (33.3)	0
**Microscopic**						
Cellularity	Low, n (%)	10 (12)	4 (10.5)	3 (9.1)	2 (66.7)	1 (16.7)
	Moderate, n (%)	52 (65)	25 (65.8)	23 (69.7)	1 (33.3)	3 (50)
	High, n (%)	18 (22.5)	9 (23.7)	7 (21.2)	0	2 (33.3)
**Cell type**	Spindle, n (%)	52 (65)	24 (63.2)	24 (72.7)	2 (66.7)	2 (33.3)
	Epithelioid, n (%)	8 (10)	5 (13.2)	1 (3.1)	0	1 (16.7)
	Mixed, n (%)	20 (25)	9 (23.7)	8 (24.2)	1 (33.3)	3 (50)
**Pattern**	Fascicles, n (%)	63 (78.7)	31 (38.7)	30 (37.5)	1 (33.3)	2 (33.3)
	Palisading, n (%)	10 (12.5)	8 (80)	1 (10)	0	1 (10)
	Storiform, n (%)	8 (10)	3 (40)	5 (60)	0	0
	Sheets, n (%)	12 (15)	4 (33.3)	5 (41.6)	2 (16.6)	1 (8.3)
**Mitosis**	<5/50HPF, n (%)	40 (50)	18 (47.4)	20 (60.6)	1 (33.3)	1 (16.7)
	>5/50HPF, n (%)	40 (50)	20 (52.6)	13 (39.4)	2 (66.7)	5 (83.3)
**Cellular atypia**	Mild, n (%)	37 (46.3)	16 (42.1)	17 (51.5)	1 (33.3)	2 (33.3)
	Moderate, n (%)	35 (43.7)	19 (50)	12 (36.4)	1 (33.3)	3 (50)
	Severe, n (%)	8 (10)	2 (5.3)	4 (12.1)	1 (33.3)	1 (6.7)
**Lymph node metastasis**		11 (13.7)	2 (18.2)	6 (54.6)	2 (18.2)	1 (9.1)
**Distant metastasis**		2 (2.5)	1 (50)	0	0	1 (50)
**Lymph node metastasis**		11 (13.7)	2 (18.2)	6 (54.6)	2 (18.2)	1 (9.1)
**Immunohistochemistry**						
CD117		75 (93.8)	36 (94.7)	30 (84.8)	3 (100)	6 (100)
DOG1		73 (91.3)	38 (97.4)	28 (84.8)	3 (100)	4 (66.7)
CD34		48 (60)	29 (73.7)	15 (42.4)	1 (33.3)	3 (33.3)
SMA		32 (40)	15 (26.3)	14 (27.3)	0	3 (33.3)
Desmin		13 (16.2)	6 (15.8)	3 (6.1)	1 (33.3)	3 (33.3)
S100		29 (36.2)	17 (36.8)	9 (24.2)	0	3 (16.7)

### Mutational Analysis

Overall KIT and PDGFRA mutations were present in 47 cases (58.8%).

**Figure 1:**
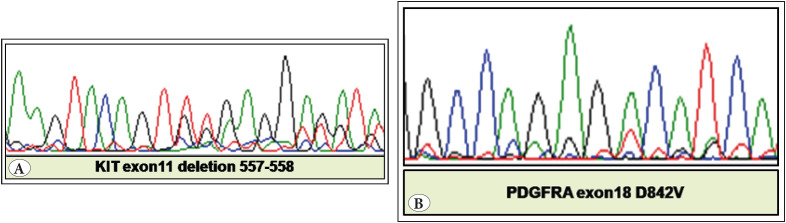
**A)** Electrophoretogram displays KIT exon 11 deletion in codons 557, 558. **B)** Electrophoretogram displays point mutation in codon 842 of PDGFRA exon 18.

*KIT exon 11 mutations* were seen in 30 (63.8%) cases with simple deletions in 17 (56.7%), point mutations in 10 (33.3%) and complex mutations (deletions and duplication/insertions) in 3 (10%) cases. Codon 557-558 deletions (Figure 1) were found in 15 cases (50%).

*KIT exon 9 mutations* were found in 9 cases (19%) which showed duplications Tyr502-503Asp in 4 cases and point mutations in 5 cases. These mutations were common in the small intestine (66.7%) followed by EGIST (22.2%) and stomach (11.1%). The spectrum of mutations in KIT exon 11 and 9 are summarized in Table 2.

**Table 2:** Spectrum of KIT mutations with location and immunohistochemistry.

**Age**	**Sex**	**Exon**	**Mutation**	**Locus**	**Location**	**CD117**	**CD34**	**SMA**	**S100**	**Desmin**	**DOG1**
76	M	11	Deletion	del 557-558	Stomach	P	N	P	N	N	P
53	M	11	Deletion	del 557-558	Stomach	P	P	P	N	N	P
54	M	11	Deletion	del 557-558	Small bowel	P	N	N	P	N	P
56	M	11	Deletion	del 557-558	Stomach	P	P	N	P	N	P
60	M	11	Deletion	del 557-559	Extraintestinal	P	P	N	N	N	P
50	M	11	Deletion	del 557-561	Stomach	P	N	N	N	N	P
40	F	11	Deletion	del 557-559	Stomach	P	P	P	N	P	P
62	M	11	Deletion	del 557-558	Stomach	P	P	N	N	N	P
47	M	11	Deletion	del 547-558	Stomach	P	P	N	N	P	P
57	F	11	Deletion	del 551-554	Small bowel	P	N	P	N	N	P
43	M	11	Deletion	del 556-564	Small bowel	P	N	P	N	N	P
73	M	11	Deletion	del 552-557	Stomach	P	P	P	N	N	P
51	M	11	Deletion	del 553-556	Stomach	P	P	N	N	N	P
58	M	11	Deletion	del 557-560	Stomach	P	P	N	P	N	P
35	M	11	Deletion	del 560	Small bowel	P	N	N	N	N	P
61	M	11	Deletion	del 554-557	Small bowel	P	N	N	N	N	P
45	M	11	PM	PT val560asp	Small bowel	P	N	P	P	N	P
57	M	11	PM	PT ser590aspgn	Small bowel	P	N	N	N	N	N
54	M	11	PM	PT arg588lys	Small bowel	P	P	P	P	N	P
55	F	11	PM	PT leu 576 pro	Extraintestinal	P	P	P	N	P	P
42	M	11	PM	PT try 557 gly	Stomach	P	P	P	P	N	P
47	M	11	PM	PT val 559 asp	Stomach	P	N	N	P	N	P
47	M	11	PM	PT val 559 gly	Stomach	P	P	N	N	N	P
67	M	11	PM	PT gly559pro	Stomach	P	P	N	P	N	P
49	M	11	PM	PT tyr553asn	Stomach	P	P	P	P	N	P
60	F	11	PM	PT tyr553asn	Stomach	P	P	N	N	N	P
69	M	11	Complex	complex del-ins 557-561, ins 557-558	Stomach	P	P	N	N	N	P
71	M	11	Complex	complex del-subs 557+one 556	Small bowel	P	P	N	N	N	P
57	M	11	Complex	complex ins 575-576	Extraintestinal	P	N	P	P	N	P
40	M	11	Complex	del homozygous 554-561	Stomach	P	P	N	N	N	P
49	M	9	Duplication	dup 502-503	Small bowel	P	P	N	P	N	P
54	M	9	Duplication	dup 502-503	Extraintestinal	P	N	N	P	P	P
53	M	9	Duplication	dup 502-503	Small bowel	P	P	P	N	N	P
68	M	9	Duplication	dup 502-503	Small bowel	P	P	P	P	N	P
46	F	9	PM	PT leu472isoleu	Small bowel	P	P	P	N	N	P
65	F	9	PM	PT phe504ser	Extraintestinal	P	N	P	P	P	N
38	M	9	PM	PT thr488meth	Small bowel	P	P	P	P	P	N
77	M	9	PM	PT cys491gly	Stomach	P	P	N	N	N	P
34	F	9	PM	PT thr488alan	Small bowel	P	P	N	N	N	P

*PM: Point mutation, **P:** Positive, **N:** Negative.

*PDGFRA exon 18 mutations* were present in 8 cases (17%), commonly located in the stomach (62.5%), and had epithelioid/mixed morphology. D842V mutation was seen in 1 case whereas D842E was seen in 5 cases (Figure 1). The spectrum of PDGFRA exon 18 mutations are summarised in Table 3. No mutations were found in PDGFRA exons 12 and 14.

**Table 3:** Spectrum of PDGFRA exon 18 mutations with locations and immunohistochemistry.

**Age**	**Sex**	**Mutation**	**Locus**	**Location**	**CD117**	**CD34**	**SMA**	**S100**	**Desmin**	**DOG1**
60	M	PM	PT aspt842glut	Stomach	P	N	N	N	N	P
39	M	PM	PT aspt842glut	Extraintestinal	P	P	N	N	N	N
53	M	PM	PT aspt842glut	Stomach	P	P	P	P	N	P
75	M	PM	PT aspt842glut	Stomach	N	P	N	P	N	P
47	M	PM	PT aspt842val	Stomach	P	P	P	P	P	P
80	M	PM	PT aspt842glut	Stomach	P	P	P	N	P	P
43	M	PM	PT his817pro	Small bowel	P	N	N	N	N	P
34	M	PM	PT Leu826Glu	Small bowel	P	N	N	N	N	P
60	M	PM	PT aspt842glut	Stomach	P	N	N	N	N	P

*PM: Point mutation, **P:** Positive, **N:** Negative.

### Genotype and Clinicopathological Features

Males had higher KIT exon 11 and PDGFRA mutations than females (p=0.03). GISTs with PDGFRA exon 18 mutations had smaller tumour size than cases with KIT exon 9 and exon 11 mutations (p=0.01). Nearly 67% of cases with KIT exon 11 mutations were present in the stomach that included 12 simple deletions, 7 point mutations and one complex mutation (Table 3). Overall 66.7% of KIT exon 9 mutations were found in the small intestine (p=0.04) of which codon 502-503 duplication was present in 75% of the small intestine (p=0.03). PDGFRA exon 18 mutations were mostly present in the stomach (62.5%) and all D842 point mutations were found in the gastric location as well (p=0.04). All colonic GISTs were wild type whereas all EGISTs were mutant (KIT exon 11 deletion in 2 cases, KIT exon 11 point mutation in one case, KIT exon 9 duplication/point mutation and PDGFRA exon 18 point mutation in one case each). The correlation of the GIST genotype with clinicopathological variables and risk stratification is shown in Table 4.

**Table 4:** Correlation of histopathological variables with mutation.

**Variables**		**KIT exon 11** **(n=30)(%)**	**KIT exon 9 (n=9) (%)**	**PDGFRA exon 18 (n=8) (%)**	**Wild type (n=33) (%)**	p- value*
**Gender**	Male	26 (86.6)	6 (66.6)	8 (100)	20 (60.6)	**0.03**
	Female	4 (13.4)	3 (33.4)	0	13 (39.4)	
**Location**	Stomach	18 (60)	1 (11.1)	5 (62.5)	14 (42.4)	**0.04**
	Small intestine	9 (30)	6 (66.7)	2 (25)	16 (48.4)	
	Colon	0	0	0	3 (9.2)	
	Extraintestinal	3 (10)	2 (22.2)	1 (12.5)	0	
**Tumour size**	0-2 cm	0	0	0	2 (6.1)	0.1
	2.1-5 cm	5 (16.7)	4 (44.5)	3 (37.5)	3 (9.2)	
	5.1-10cm	12 (40)	2 (22.2)	4 (50)	11 (33.3)	
	>10 cm	13 (43.3)	3 (33.3)	1 (12.5)	17 (51.5)	
**Cell type**	Spindle	19 (63.3)	7 (77.8)	3 (37.5)	23 (69.7)	0.4
	Epithelioid	2 (6.7)	0	2 (25)	3 (9.2)	
	Mixed	9 (30)	2 (22.2)	3 (37.5)	7 (21.2)	
**Cellular atypia**	Mild	18 (60)	4 (44.5)	3 (37.5)	12 (36.3)	**0.01**
	Moderate	12 (40)	5 (55.5)	2 (25)	16 (48.4)	
	Severe	0	0	3 (37.5)	5 (15.3)	
**Mitosis group**	<5/50HPF	11 (36.7)	5 (55.5)	6 (75)	18 (54.6)	0.2
	>5/50HPF	19 (63.3)	4 (44.5)	2 (25)	15 (45.4)	
**Risk group**	None	0	0	0	1 (3)	**0.01**
	Very low	1 (3.3)	0	3 (37.5)	0	
	Low	4 (13.3)	3 (33.3)	1 (12.5)	6 (18.2)	
	Intermediate	5 (16.6)	0	1 (12.5)	8 (24.2)	
	High	20 (66.6)	6 (66.7)	3 (37.5)	18 (54.5)	
**Lymph node metastasis**		2 (6.7)	1 (11.1)	1 (12.5)	7 (21.2)	0.4
**Distant metastasis**		2 (6.7)	0	0	0	0.3
**Recurrence**		6 (20)	0	2 (40)	4 (12.1)	0.3

*Pearson Chi square and Fisher Exact tests were used wherever applicable

### Genotype and Histomorphology

**Figure 2:**
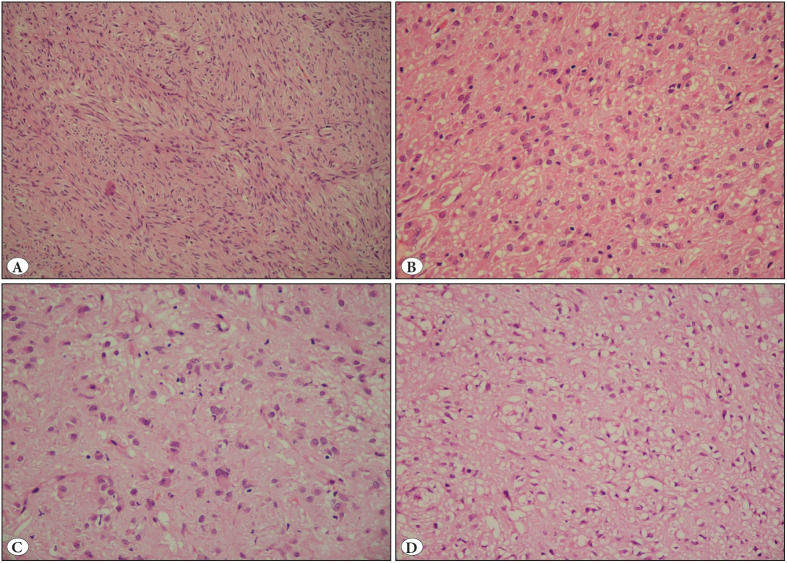
**A)** Spindled tumour cells arranged in fascicles in a KIT11 mutant GIST (H&E; x20). **B)** Tumour cells with epithelioid morphology, round nuclei, vesicular chromatin and abundant eosinophilic cytoplasm (PDGFRA mutant gastric GIST) (H&E; x20). **C)** Tumour cells with moderate cellular pleomorphism in PDGFRA mutant EGIST (H&E; x20). **D)** Tumour cells with paranuclear vacuolation in a KIT exon 9 mutant small intestinal GIST (H&E; x20)

KIT exon 11 and 9 mutations were predominantly associated with spindle cell morphology (Figure 2) compared to PDGFRA mutations that had epithelioid (Figure 2) or mixed phenotype (p=0.4). Mild to moderate nuclear atypia was significantly associated with KIT exon 11 (100%) and exon 9 mutations (100%) whereas 37.5% cases with PDGFRA exon 18 mutation and 15% of wild type GISTs showed severe atypia including plasmacytoid cells and binucleate tumour giant cells (p=0.01) (Figure 2).

A diffuse sheet-like pattern was seen in 10 cases; 6 of which harbored KIT exon 11 deletions and 4 were wild type. All cases with substitutions and insertions had fascicles and/or a palisaded pattern. Paranuclear vacuolization was commonly observed in cases with KIT exon 9 mutations (Figure 2). It may be assumed that cases with both KIT exon11 deletions and wild type have an aggressive course with high cellularity and hence have a diffuse sheet-like pattern.

### Genotype and Survival

**Figure 3:**
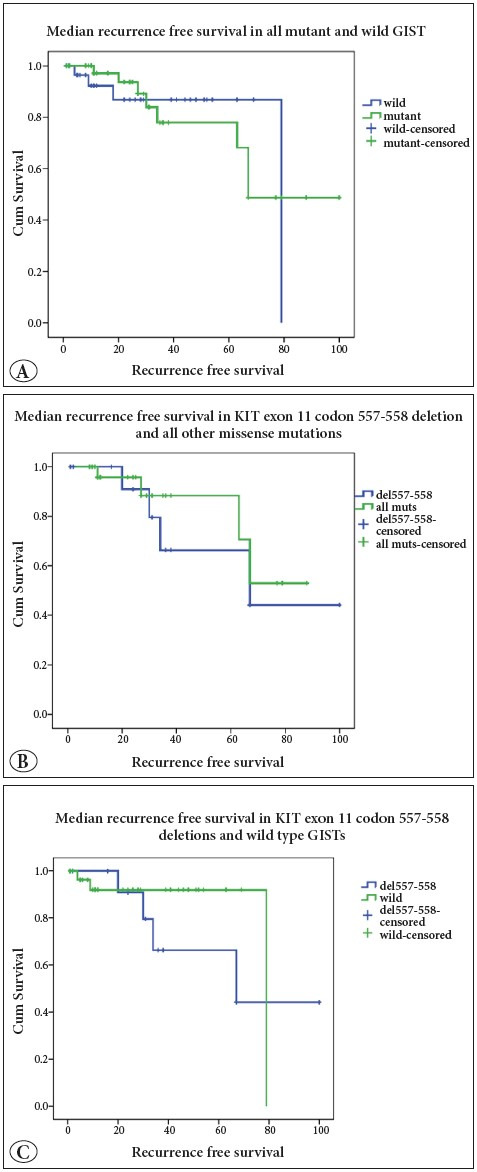
Survival curves: **A)** Wild and mutant GISTs. **B)** KIT exon 11 codon 557-558 deletion and all other mutant GISTs. **C)** KIT exon 11 codon 557-558 deletion and wild GISTs.

Follow-up was available in 73 patients with median follow-up of 27 and mean follow-up of 34.2 months ± 26.7 (range: 1-101 months). Recurrence was observed in 12 patients (9 males, 3 females) within 6 to 34 months post-surgery in 9 high risk, 2 intermediate risk and 1 low malignant risk cases. Six recurrent cases (50%) showed KIT exon 11 mutation with 4 cases (33.4%) having codon 557-558 deletion and two (16.7%) having point mutations. Median recurrence free survival (RFS) in patients with wild type GIST was higher than in patients with mutated GIST (79 months vs. 67 months, respectively; p=0.9) (Figure 3). The 5-year RFS in patients with KIT and PDGFRA wild type was also higher (87% vs.48%) than in mutant GIST (p=0.6). The median RFS in patients with codon 557-558 deletion was 67 months compared to 84 months in patients with all other missense mutations (p=0.6) (Figure 3). The 5-year RFS was 42% in patients with codon 557-558 deletion and 53% in other missense mutations (p=0.7) of KIT 11. The codon 557-558 deletion also showed decreased median RFS of 68 months (5-year survival: 42%) compared to wild type GISTs with RFS of 79 months (5-year survival: 92%) (p=0.4) (Figure 3).

## DISCUSSION

GISTs are mesenchymal tumours with malignant risk potential ranging from very low to high risk. The diagnosis of GIST relies on a panel of immunohistochemical markers whereas genotyping of GIST is essential for dosage and predicting the response of tyrosine kinase inhibitors. Since GISTs harbour various mutations having a wide range of frequency with their available targets, it is prudent to prioritize genetic mutation in this disease and use the resources judiciously, especially in developing countries.

In the present study, tumour size ranged from 1.5-30 cm with a mean of 10.3 cm, with larger tumours in large intestinal (mean: 17 cm) and extraintestinal location (mean: 12.9 cm) compared to the stomach and small intestine (mean-9.8 cm). In the series by Miettinen et al., the median size of gastric GISTs was 6 cm (9) and several European series have reported a median size of 5-5.6 cm (2-10 cm) (10,11). On the contrary, median tumour size from other Indian series varies from 10.6 to 10.9 cm (12,13), similar to our series which could be related to later admission to the hospital at an advanced symptomatic stage.

Mixed and epithelioid cell types are usually encountered in a gastric location (14). Interestingly, in the present study 50% of our EGISTs also showed a mixed cell type and this has not been observed in the EGIST of other series (9,10).

High concordance (87.5%) was observed between CD117 and DOG1 immunoreactivity; however, four of five (80%) CD117 negative cases stained for DOG1. Foo et al. have reported one third of CD117 negative cases to be positive for DOG1 (15).

KIT/PDGFRA mutation frequency was lower in the present study compared to western data. This may be explained by ethnical variation which is well known for lung and colorectal cancers (16). Although genotyping studies from India and other Asian countries are fewer, the series from Taiwan and China also showed similar lower rate of mutations in KIT and PDGFRA genes (17–19). In our cases, KIT exon 11 and PDGFRA exon 18 mutations were commonly seen in the stomach whereas KIT exon 9 mutations were seen in the small intestine, similar to other studies (20,21). However, Daniel et al. showed KIT 11 deletions to have different locations having a wide spectrum of morphology (22). A peculiar finding noted in this study was the presence of 100% mutation in EGISTs.

Male gender had higher rates of both KIT11 and PDGFRA mutations as well as wild genotype as compared to females (p=0.03), indicating that all kinds of GIST genotype is possible in males (20). Lv et al. showed that all mutations were more common and associated with poor RFS in males (23). PDGFRA mutation showed a significant correlation with male gender in the present study (p=0.02), similar to Daniels et al.; however, KIT 9 mutation was equally present among both sexes (22). PDGFRA exon 18 mutations had smaller tumour size than cases with KIT exon 9 and 11 mutations (p=0.01), similar to the series of Wozniak et al. (20).

Agaimy et al. and Daum et al. noted that epithelioid or mixed cell morphology, presence of mast cells, multinucleated giant cells and myxoid stroma were more frequently associated with PDGFRA mutation (7,24). We also observed similar findings where 5 of 8 PDGFRA mutated cases had epithelioid morphology and two cases had plasmacytoid cells and multinucleated cells. Unfortunately, SDH, KRAS and BRAF mutations could not be performed in KIT-PDGFRA wild type cases due to financial constraints, which is one of the major limitations of this study.

Morphological predictors of prognosis or RFS were mitotic activity (p=0.009), tumour necrosis (p=0.04), and cellular atypia (p=0.03).

### Suggested Algorithm for Molecular Testing in GIST Based on Morphological Features

**Figure 4:**
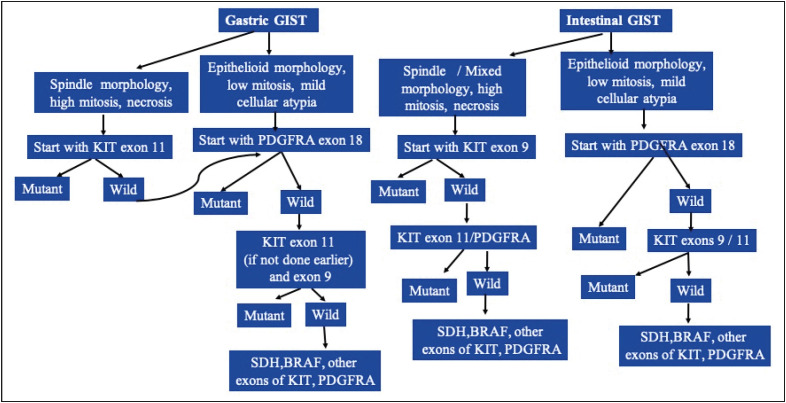
The figure shows suggested algorithm for molecular testing in GIST.

Considering all histomorphological or genotypical features observed in the present study, we can argue that certain morphological features can be associated with a particular genotype. Therefore, we can suggest that the sequence of testing can be modified especially for patients/centres where financial affordability for multiple investigations is a concern (Figure 4). As most of the GIST mutations are mutually exclusive, the order of mutation testing can be devised for each set of morphological features. Tumours in the gastric location with spindle cell morphology and high mitosis may be tested for KIT exon 11 while gastric tumours with low mitosis, epithelioid morphology and low cellular atypia may be tested initially for PDGFRA exon 18 in the first phase, or if feasible, gastric GISTs should be first tested for KIT exon 11 followed by PDGFRA exon 18. If both are wild type then one may proceed with KIT exon 9 testing. About 20-40% of double negative GISTs are positive for SDH mutations and another 15% of triple negative (KIT, PDGFRA, SDH negative) cases harbour mutations in RAS/BRAF (25,26), which may be checked if the above mutations are negative. SDH immunohistochemistry can be done either following or in conjunction with KIT exon 11 and 9 mutation testing. Later, the BRAF, KIT exons 13, 17 and PDGFRA exon 12 mutations may be tested, which account for <5% of all mutations. Most SDH deficient GISTs have characteristic dumb bell/lobulated shape with thick fibrous bands, epithelioid morphology and frequent lymph node metastasis; SDH testing may precede KIT exon 11 mutation testing for suspected cases. It should be noted that this suggested algorithm is suited mostly for resource limited situations, and does not disagree with studying multiple mutations if laboratory resources and the patient’s status permits.

In conclusion**, **this study is dedicated to the present association of morphological features with mutations occurring in GIST. Genotyping GIST is becoming mandatory as it is recommended to predict dosage and response to imatinib therapy. The only matter of concern is its availability and affordability for the individual patient. Since GISTs harbor multiple mutations, next generation sequencing (NGS) is the most suitable technology today to study mutations. However, the biggest limitations with NGS are the cost and the necessary expertise which is not available in all centres. Therefore, most of the laboratories have to depend on the gold standard Sanger sequencing and it is always prudent to prioritize the mutation testing especially in settings with limited financial means. We suggest testing initially for KIT exon 11 and/or PDGFR exon 18 in gastric GISTs depending on spindle cell and epithelioid or mixed cell morphology respectively, followed by KIT exon 13, 17 and PDGFRA exon12.

## Conflict of Interest

The authors declare no conflict of interest.
